# Learning new surgical techniques in low and middle income countries, approval processes, and the impact of artificial intelligence

**DOI:** 10.3389/fsurg.2025.1647899

**Published:** 2025-08-08

**Authors:** Long Cong Duy Tran, Helal Metwalli, Dat Tien Le, Samuel Amo-Afful, Mario Salib Todry Gerges, Hajer Hatim Hassan Ahmed, Dinh Thi Kim Quyen, Abdelrahman Gamil Gad, Phillip Tran, Nguyen Tien Huy

**Affiliations:** ^1^ Department of Hepatobiliary and Pancreatic Surgery, University Medical Center at Ho Chi Minh City, Ho Chi Minh City, Vietnam; ^2^ Department of Surgery, Faculty of Medicine, University of Medicine and Pharmacy at Ho Chi Minh City, Ho Chi Minh City, Vietnam; ^3^ Faculty of Human Medicine, Benha University, Benha, Egypt; ^4^ Online Research Club, Nagasaki, Japan; ^5^ Queen Alexandra Hospital, Portsmouth, United Kingdom; ^6^ Al Mokattam Insurance Hospital, Cairo, Egypt; ^7^ Faculty of Medicine, Alzaiem Alazhari University, Khartoum, Sudan; ^8^ Pediatric Surgery Department, University of Medicine and Pharmacy at Ho Chi Minh City, Ho Chi Minh City, Vietnam; ^9^ Russell H. Morgan Department of Radiology and Radiological Science, Johns Hopkins University School of Medicine, Baltimore, MD, United States; ^10^ Invasive Cardiology Nam Can Tho University, Can Tho, Vietnam; ^11^ Institute of Research and Development, Duy Tan University, Da Nang, Vietnam; ^12^ School of Medicine and Pharmacy, Duy Tan University, Da Nang, Vietnam; ^13^ School of Tropical Medicine and Global Health (TMGH), Nagasaki University, Nagasaki, Japan

**Keywords:** surgery, artificial intelligence, LMIC (low and middle income countries), healthcare, residency accreditation

## Abstract

Training in surgery and approval of new techniques in low- and middle-income countries (LMICs), usually depends on informal apprenticeship systems, that is often lacking standardization, structured mentorship and produce inconsistent patient outcomes. These challenges are particularly severe in rural areas, where training opportunities and healthcare infrastructure are limited. Recently, artificial intelligence (AI) has emerged as a reliable solution, providing applicable, Quantitative methods for skill development, competency evaluation and regulatory supervision. AI-powered tools, such as virtual reality (VR) simulations and tele-mentoring platforms, provide independent skill assessments and expand access to high-quality surgical education. However, implementing AI in LMICs faces some challenges, including inadequate resources, financial constraints and ethical issues related to data security and Equitable algorithms. This review compares usual surgical training and approval processes in LMICs and evaluates the promising role of AI to fill existing gaps and compares both approaches in terms of applicability, cost-effectiveness and impact on patient outcomes.

## 1 Introduction

Implementing new surgical techniques, in Low and Middle -Income Countries (LMICs), often follows informal, apprenticeship-style pathways. Training usually runs through bedside observation, short-term workshops, or mentorship from visiting specialists. These approaches lack standardized curricula and objective assessment tools. This system can lead to wide variability in surgical competency among surgeons and patient outcomes, especially in rural areas where access to mentorship and infrastructure is limited (1). Despite the absence of strong, evidence-based validation, the successful application of a novel technique by a single experienced surgeon can be sufficient to gain institutional or even ministry-level approval for broader use (2). Recently, artificial intelligence (AI) has begun to reshape rules for surgical education in LMICs. AI-powered tools, particularly those using computer vision and machine learning, offer data-driven solutions for skill acquisition and performance assessment. These technologies can analyze surgical videos and kinematic data, to distinguish between expert and limited performance, providing standardized and reliable assessments (3, 4). AI platforms also enable personalized feedback and remote simulation-based learning, allowing trainees to practice and improve skills outside traditional settings (5). These new technologies are impactful in LMICs, where training opportunities are sparse and surgical capacity building is a critical need (1).

This review explores the traditional processes by which new surgical techniques are learned and approved in LMICs, examines how AI addresses existing limitations and compares these pathways to highlight their effects on surgical quality, information access and patient safety.

## 2 Traditional training and approval in LMICs

### 2.1 Learning new techniques

The junior surgeons observe and assist their seniors in the operating room, then gradually practice. Mentorship system is still the base for surgical education, However, the shortage of experienced mentors, especially for advanced operations like laparoscopy or endoscopy, minimizes opportunities, especially in rural areas. The development of new surgical skills in LMICs is constrained by limited access to formal training programs, which are often concentrated in urban centers of high-income countries (HICs). Some surgeons pursue international fellowships in HICs, but high costs, visa restrictions and limited availability make this option feasible for only a few. Short-term workshops and cadaver labs provide concentrated exposure to new techniques, yet their sporadic nature and high costs limit scalability. Industry-sponsored training, often tied to proprietary technologies like laparoscopic systems, offers another avenue, but these programs prioritize commercial interests and lack standardization. These fragmented approaches result in inconsistent skill acquisition, leading to variable patient outcomes and potential risks to patient safety (6, 7).

### 2.2 Gaining approval without prior experience

Approval systems in Many LMICs, lack standardized criteria to assess surgeon clinical qualifications, relying instead on Preliminary evaluations by senior colleagues or hospital committees. Approving of new surgical techniques in LMICs is often informal and ethically complex. In some cases, surgeons perform a small number of demonstration cases under supervision, but access to experienced assessors is limited. Ministries of Health may grant approvals based on limited evidence, such as case reports or international guidelines, which may not account for local patient demographics or resource constraints. Certificate-based credentialing, where completion of a workshop or simulated-skills course suffices for operative privileges, is common but often lacks objective proof of proficiency. These regulatory gaps raise concerns about patient safety, as inexperienced surgeons may undertake complex procedures without adequate preparation, potentially compromising informed consent and clinical outcomes (8–10).

## 3 AI-Enhanced training and regulatory frameworks

### 3.1 AI in surgical training

Due to the lack of professional surgical mentorship in LMICs, there has been development of alternative solutions such AI platforms such as Touch Surgery which utilises virtual or augmented reality. Among several AI benefits include identifying errors, tracking performance, real-time objective feedback and steps for improvements. This enhances personalised surgical training tailored to the learner's current skill level leading to faster and efficient advancement. Furthermore, learners can receive real-time remote supervision from seniors or experts through digital technologies such as tele-mentoring.

All this have reduced barriers such as geographical location and unavailable experienced expert support. The result culminating into high-quality surgical education with less expenses on infrastructural constraints or inadequate in-person mentors (11, 12).

### 3.2 AI in credentialing and approval

Surgical proficiency among Surgical residents can be assessed with the Objective Structured Assessment of Technical Skills (OSATS). Machine learning models, trained on surgical videos, analyze performance metrics like speed, precision and error rates, providing standardized assessments that overcome the subjectivity of traditional evaluations. These advancements streamline approvals and enhance patient safety by ensuring only competent surgeons perform new techniques. Through analysis of simulation and real-operation data, predictive metrics identifies surgeons' capability for independent practice, providing assessors with unbiazed decision. For example, after credentialing, AI systems enable continuous quality monitoring by reviewing recorded procedures to detect technique drift or rising complication rates, triggering targeted remedial training or privilege reassessment. Some regulators are exploring automated certification systems, where AI-verified training logs detailing simulated cases, competency scores and error trends are submitted as part of credentialing packages. AI enhances the objectivity and rigor of credentialing and regulatory processes (13–15), Table 1 shows comparison between traditional and AI enhanced pathways.

**Table 1:** Comparison between traditional vs. AI-enhanced pathways.

Aspect	Traditional pathway	AI-enhanced pathway
Training delivery	Relies on short in-person workshops and ad-hoc proctorship, limited by geography and mentor availability	Combines AI-driven VR/AR simulation with remote tele-mentoring, enabling scalable and accessible training
Competency assessment	Depends on subjective sign-off by senior colleagues or certificate of workshop attendance	Uses objective AI-derived metrics and standardized proficiency benchmarks for consistent evaluation
Regulatory approval	Based on limited demonstration cases and ministry sign-off with minimal data	Requires submission of AI-verified simulation logs and tele-mentored case records for evidence-based approvals
Ongoing oversight	Involves periodic audits and self-reported outcomes, with limited systematic monitoring	Employs continuous AI monitoring of recorded procedures, with automated alerts for deviations or complications
Scalability	Constrained by resource availability, mentor shortages, and logistical barriers	Highly scalable through virtual platforms and low-bandwidth solutions, reaching rural and underserved areas
Cost	High for international fellowships and workshops, with limited long-term investment	High initial investment for AI infrastructure, but cost-effective over time with scalable platforms
Ethical considerations	Risks patient safety due to informal approvals and variable training quality	Enhances safety through rigorous assessments but raises concerns about data privacy and algorithm bias
Patient safety	Higher risk during early cases due to limited practice and oversight	Reduced risk through simulation-based practice and predictive analytics for complication prevention

### 3.3 Challenges of AI adoption in LMICs

LMICs are burdened with several mitigating factors to implement AI in surgical training or care. Among many include poor internet access, unstable electricity and technical support. Furthermore, the overall cost in hardware, software license and system updates demand enormous initial investments and can be achieved through innovative funding models such as public-private collaborations or international grants. There also exists the challenge of data or information governance involving how surgical videos and performance metrics are handles under patient privacy laws, and the complexities involving data ownership ambiguities.

Lastly, some regulators and professionals push back the implementation of AI platforms particularly because of familiarity with conventional methods. There have also been some concerns about the accuracy and transparency of AI's decision-making process (16–18).

This expanded comparison illustrates how AI shifts surgical training and approval from resource-intensive, subjective processes to scalable, data-driven systems, improving equity, safety and regulatory rigor.

## 4 Future directions and recommendations

The future of surgical training within LMICs lies between the mix of hybrid educational models that balances traditional in-person and AI driven simulation platforms. This would allow dual needs for scalability and practical experience acquisition (19, 20). To ensure a sustainable future for AI integration in LMICs surgical training, it is important that stakeholders such as educators, private sectors and policy makers work in partnership to provide and fund affordable, locally sensitive context AI platforms within their region (21, 22). International collaborations and integration into existing healthcare education budgets, with cost-benefit perspective can overcome the financial constraints particularly taking into consideration the long-term investment.

AI incorporation into surgical training in LMICs would require enhances in information governance systems that aligns with ethical standards and objective validation (23, 24).

### 4.1 Standardization of AI-based surgical training

Because of limited resources in LMICs, healthcare centers can vary in their needs even within the same country. So, we recommend the development of centralized guidelines, shared evaluation metrics and partnerships with regional medical councils. And these guidelines should be accredited by higher healthcare authorities like ministries of health in these countries. Which will ensure training equivalency across centers. These strategies will help to standardize the surgical skills acquired within the all centers.

### 4.2 Data privacy and bias in AI models

Data privacy is a major concern when talking about AI models, particularly in LMICs where there are inconsistent data standards, unclear policies, fragmented data architectures, financial constraints and confidentiality breaches (25). As a response to the growing use of AI in healthcare, several universities are working on developing a secure access to AI model to protect sensitive data. For example, Johns Hopkins University has created Hopkins AI (26) which provides access to several AI models including OpenAI, Meta and Claude while protecting sensitive patient data from being leaked. Several methods can be used to anonymize data used in AI models like, replacing protected health information (PHI) with codes and avoiding the use of any identifiers on AI models. Adherence to local protective laws and regulations will be very helpful in protecting data privacy when using AI models as well. Bias in AI models poses a major threat to the fairness and effectiveness of AI-driven surgical training. These systems are only as good as the data they learn from. When AI is trained predominantly on data from high-income countries, it may fail to accurately assess skills or predict outcomes in low-resource settings. This can result in unfair evaluations, misclassification of surgeon competence and inappropriate training recommendations. Bias can stem from unbalanced datasets, lack of contextual diversity or developer assumptions. To reduce bias, AI models must be trained on inclusive, representative datasets and undergo validation across diverse geographic, cultural and clinical environments. Testing multiple models and comparing results to guidelines and literature is also crucial in avoiding bias produced by AI models.

Finally, launching pilot programs in a range of urban and rural LMIC settings is essential. These efforts should systematically evaluate how AI integration influences skill development, surgical outcomes and regulatory preparedness, generating the evidence base required for broader implementation and policy integration (27, 28).

## 5 Conclusion

In LMICs, surgical training and credentialing traditionally rely on informal, mentor-based systems with limited oversight and variable outcomes. This system, while accessible, often compromises patient safety and lacks standardized competency assessment. The integration of artificial intelligence offers a transformative shift, providing scalable, objective and data-driven solutions for training, evaluation and regulatory processes. AI-driven platforms enhance access to high-quality education through simulation and tele-mentoring while enabling continuous monitoring and evidence-based credentialing. Despite infrastructural and ethical challenges, hybrid models combining AI and conventional mentorship may present the most viable path forward. To ensure effective adoption, investment in digital infrastructure, governance frameworks and capacity building is essential. Ultimately, AI holds significant potential to elevate surgical standards, improve equity in care and support safer, more accountable surgical practices across resource-limited settings.
